# Epidemiological and Clinical Features of Severe Fever with Thrombocytopenia Syndrome in Japan, 2013–2014

**DOI:** 10.1371/journal.pone.0165207

**Published:** 2016-10-24

**Authors:** Hirofumi Kato, Takuya Yamagishi, Tomoe Shimada, Tamano Matsui, Masayuki Shimojima, Masayuki Saijo, Kazunori Oishi, SFTS epidemiological research group-Japan

**Affiliations:** 1 Feild Epidemiology Training Program (FETP), National Institute of Infectious Diseases, Shinjuku, Tokyo, Japan; 2 Division of Global Infectious Diseases, Department of Infection and Epidemiology, Graduate School of Medicine, Tohoku University, Sendai, Miyagi, Japan; 3 Infectious Disease Surveillance Center, National Institute of Infectious diseases, Shinjuku, Tokyo, Japan; 4 Department of Virology I, National Institute of Infectious diseases, Shinjuku, Tokyo, Japan; University of Minnesota College of Veterinary Medicine, UNITED STATES

## Abstract

Although severe fever with thrombocytopenia syndrome (SFTS) was first reported from Japan in 2013, the precise clinical features and the risk factors for SFTS have not been fully investigated in Japan. Ninety-six cases of severe fever with thrombocytopenia syndrome (SFTS) were notified through the national surveillance system between April 2013 and September 2014 in Japan. All cases were from western Japan, and 82 cases (85%) had an onset between April and August. A retrospective observational study of the notified SFTS cases was conducted to identify the clinical features and laboratory findings during the same period. Of 96 notified cases, 49 (51%) were included in this study. Most case-patients were of advanced age (median age 78 years) and were retired or unemployed, or farmers. These case-patients had a history of outdoor activity within 2 weeks before the onset of illness. The median serum C-reactive protein concentration was slightly elevated at admission. Fungal infections such as invasive aspergilosis were found in 10% of these case-patients. Hemophagocytosis was observed in 15 of the 18 case-patients (83%) whose bone marrow samples were available. Fifteen cases were fatal, giving a case-fatality proportion of 31%. The proportion of neurological abnormalities and serum concentrations of lactate dehydrogenase and aspartate aminotransferase were significantly higher in the fatal cases than in the nonfatal cases during hospitalization. Appearance of neurological abnormality may be useful for predicting the prognosis in SFTS patients.

## Introduction

Severe fever with thrombocytopenia syndrome (SFTS) is a tick-borne infectious disease caused by SFTS virus (SFTSV), which is a newly identified bunyavirus in the genus *Phlebovirus* of the family *Bunyaviridae* [1]. SFTS was first reported from Central and Northeast China in 2009 [2], and about 2,500 cases were reported from 11 provinces in China in 2010–2012 [3]. SFTS is considered endemic to Japan and South Korea based on the identification of SFTS patients in 2013 [4,5].

SFTSV is found in ticks such as *Haemaphysalis longicornis* in China and South Korea [6,7], and *Amblyomma testudinarium* and *Ixodes nipponensis* in South Korea [7], which indicates the potential vectors of SFTSV. In Japan, SFTSV has been recently detected in several species of ticks including *A*. *testudinarium* and *H*. *longicornis* (Morikawa S. Personal communication). Human-to-human transmission of SFTSV through close contact with blood and/or body secretion of patients has been reported [8–11].

After the first SFTS patient was identified in Yamaguchi prefecture, western Japan, in 2013 [4], an interim case definition was introduced to implement ad hoc surveillance, including retrospective case detection, to target primarily hospitalized patients in Japan [12]. Since March 3, 2013, physicians have been legally required to notify all confirmed cases of SFTS to their local public health center immediately after diagnosis, and the local public health center then reports the case information to the Ministry of Health, Labour and Welfare through the national surveillance system, National Epidemiological Surveillance of Infectious Diseases (NESID), in compliance with the Infectious Diseases Control Law. Subsequently, 161 confirmed SFTS cases were reported to the NESID up to December 2015. Although demographic data related to these SFTS cases have been reported to the NESID, these data were insufficient to demonstrate the precise clinical features and the risk factors for SFTS. We therefore conducted a retrospective observational study to identify the precise epidemiological and clinical features of SFTS in Japan.

## Methods

### Study Design and Data Collection

The physicians who notified NESID about all cases of SFTS with onset between March 1, 2013 and September 30, 2014 were asked to participate in this study by completing a questionnaire sent by postal mail. Demographic data, social history, history of outdoor activity 2 weeks before the onset of illness, clinical symptoms, and laboratory data of the SFTS patients were collected through the questionnaires. The physicians who agreed to participate in this study collected the information about each patient from medical charts or direct interview with the case-patient or a family member of the case-patient by telephone after obtaining informed consent from the case-patient and/or family member. Clinical information and laboratory data during the acute phase within 2 weeks after the onset of illness were collected. Basic demographic data about each SFTS patient during the study period were extracted from the NESID database.

Proven invasive aspergillosis was diagnosed by histopathological findings of Grocott’s stained lung tissue, and probable invasive aspergillosis was diagnosed by an infiltrative shadows on chest computed tomography and positive galactomannnan result [13]. Oral candidiasis was diagnosed by typical clinical appearance of mucosal membrane.

### RT-PCR for SFTSV

For all cases reported to the NESID, the laboratory confirmation of SFTSV infection was performed using reverse transcription PCR (RT-PCR) in the local public health institute and/or the National Institute of Infectious Diseases (NIID), as described previously [14]. In brief, the total RNAs were extracted from blood specimens. SFTSV genome-specific primer sets for detecting nucleoprotein region were selected, and reverse transcription step was performed under provided condition. After electrophoresis on 1% agarose gels, the PCR products with the expected sizes were visualized by staining with GelRed (Biotium, Hayward, CA). The positive-control plasmid, which provides 584 bp or 587 bp of PCR products by primer sets 1 or 2, respectively, was subjected to RT-PCR simultaneously with the specimens.

### Inclusion and exclusion criteria

Case-patients who presented any symptoms of fever and gastrointestinal symptoms or any laboratory findings including thrombocytopenia (<10.0 × 10^4^/μL), leucopenia (<4000 cells/μL), and elevated levels of liver enzyme and were confirmed by laboratory examination for SFTSV infection. Case patients who were not confirmed by laboratory examinations or those caused by other etiologies were excluded.

### Statistical Analysis

We used Fisher’s exact or the Wilcoxon rank-sum test to compare the characteristics between fatal and nonfatal cases. All p values were two-sided, and p<0.05 was considered significant. All data were analyzed using STATA 13^®^ software for Windows (StataCorp LP, College Station, TX, USA).

### Ethical Statement

The research protocol was approved by the research and ethics committees of NIID (no.549). This study was carried out according to the principles expressed in the Declaration of Helsinki The patient or guardian provided written informed consent for all cases.

## Results

### Basic Characteristics

Ninety-six cases were reported to NESID by 72 physicians during the study period. Thirty-six of the 72 physicians agreed to participate in this study. All cases were reported from the prefectures in western Japan (S1 Fig and S1 Table). All cases were diagnosed by RT-PCR; no case was diagnosed by serological assays. The characteristics of the 96 case-patients are shown in S2 Table. The overall incidence of SFTS was 0.05 cases/100,000 person-years during the study period. Most cases (82%) had an onset of disease between May and August in 2013 (n = 28) and between April and August in 2014 (n = 51) (S2 Fig).

The data for 49 of 96 cases (51%) were included in this study. The baseline characteristics of the case-patients are shown in Table 1. Of the 49 included cases, 21 (43%) and 28 (57%) were reported in 2013 and 2014, respectively. The numbers of total cases/ fatal cases for the regions were; respectively 22/ 6 in the Shikoku-region, 18/ 6 in the Kyusyu-region, 7/ 2 in the Chugoku-region, and 2/ 1 in the Kinki-region. The median age of case-patients was 78 years (interquartile range [IQR]: 65–84), and 32 (65%) of the case-patients were women.

**Table 1:** Basic characteristics of 49 case-patients with severe fever with thrombocytopenia syndrome in Japan.

	Total (n = 49)	Nonfatal (n = 34)	Fatal (n = 15)	p-value
Sex				
Male	17 (35%)	12 (71%)	5 (29%)	
Female	32 (65%)	22(69%)	10(31%)	
Age, y [IQR]	78 [65–84]	69 [62–78]	83 [79–87]	0.0033*
40–49	2 (4%)	2 (6%)	0 (0%)	
50–59	6 (12%)	5 (15%)	1 (7%)	
60–69	11 (22%)	11 (32%)	0 (0%)	
70–79	10 (20%)	6 (18%)	4 (27%)	
80–89	17 (35%)	9 (26%)	8 (53%)	
90–99	3 (6%)	1 (3%)	2 (13%)	
Underlying medical condition				
Hypertension	23 (47%)	16 (47%)	7 (47%)	1.00^†^
Diabetes	12 (24%)	8 (24%)	4 (27%)	1.00^†^
Cancer	2 (4%)	1 (3%)	1 (7%)	0.52^†^
Time from onset to initial visit, d [IQR]	3 [2–5]	3 [3–5]	4 [1–6]	0.61*
Time from onset to admission, d [IQR]	4 [2–5]	4 [4–5]	4 [1–6]	0.69*
Time from onset to death, d [IQR]	–	–	8 [5–11]	
Entering an ICU	14 (29%)	7 (21%)	7 (47%)	0.09^†^

Unless indicated otherwise, the data are expressed as number (percentage).

Note: Abbreviations. y, year; IQR, interquartile range; d, day; ICU, intensive care unit.

*Wilcoxon rank-sum test

†Fisher’s exact test

Fifteen cases were fatal, which produced a high case-fatality proportion of 31%. The fatal case-patients were significantly older than the nonfatal case-patients (p = 0.0033). No significant difference was found in age (p = 0.4589), in sex (p = 0.1050) or in case-fatality proportion (p = 0.9299) between the case-patients who were included in this study (n = 49) and those who were reported to NESID, but not included in this study (n = 47).

Of the 49 case-patients included in this study, 29 (59%) were unemployed or retired, 10 (20%) were farmers, 2 (4%) were housewives, and 8 (16%) were classified into other employment categories. The recent outdoor activities before the onset of illness included farming for 32 case-patients (65%), plant collection for 5 (10%), forestry for 5 (10%), and hunting for 2 (4%). Underlying disease was reported in 31 case-patients (65%). Twenty-three (47%), 12 (24%), and 2 (4%) had hypertension, diabetes mellitus, and cancer, respectively.

One of 49 case-patients with SFTS was excluded from the subsequent analysis of clinical data because this person was not hospitalized. The following characteristics were analyzed based on the data of the 48 case-patients who were hospitalized. The median time from the onset of disease to the initial hospital visit was 3 days (IQR: 2–5). The median time from the onset to admission was 4 days (IQR: 2–5), and this period did not significantly differ between the fatal and the nonfatal cases (p = 0.61, p = 0.29, respectively). The median time from the onset to death was 8 days (IQR: 5–11).

### Clinical Characteristics

The clinical characteristics of the 48 hospitalized case-patients at the time of admission are shown in Table 2. Most case-patients developed fever (94%). Traces of tick bite were found in only 21 case-patients (44%). Gastrointestinal symptoms such as diarrhea (71%), abdominal pain (38%), and nausea (29%) were frequent at admission in these case-patients. Neurological findings, such as disorientation (42%), muscle weakness (21%), tremor (8%), and seizure (2%), were also observed in these case-patients. The signs and symptoms at admission did not differ between the fatal and nonfatal cases (Table 2). By contrast, the percentages of patients exhibiting disorientation (23%), seizure (15%), and tremors (13%) were significantly higher in the fatal cases than in the nonfatal cases; the risk ratios were 1.94 for disorientation (p = 0.007), 6.63 for seizure (p = 0.008), and 3.46 for tremor (p = 0.04) during the hospitalization (Table 3).

**Table 2:** Clinical characteristics of 48 case-patients with severe fever with thrombocytopenia syndrome at admission grouped according to outcome in Japan.

	At admission					
	Total (n = 48)	Nonfatal (n = 33)	Fatal (n = 15)	RR	95% CI	p-value*
Temperature ≥38°C	43 (90%)	29 (88%)	14 (93%)	1.06	0.88–1.28	1.00
Fatigue	40 (83%)	27 (82%)	13 (87%)	1.11	0.99–1.25	0.54
Gastrointestinal symptoms	38 (79%)	26 (79%)	12 (80%)			
Diarrhea	34 (71%)	24 (73%)	10 (67%)	0.98	0.66–1.45	1.00
Abdominal pain	18 (38%)	14 (42%)	4 (27%)	0.72	0.31–1.73	0.50
Nausea	14 (29%)	10 (30%)	4 (27%)	0.88	0.33–2.34	1.00
Neurological symptoms	25 (52%)	15 (45%)	10 (67%)			
Disorientation	20 (42%)	11 (33%)	9 (60%)	1.64	0.87–3.06	0.20
Muscle weakness	10 (21%)	7 (21%)	3 (20%)	1.17	0.37–3.84	1.00
Tremor	4 (8%)	1 (3%)	3 (20%)	6.92	0.79–60.49	0.08
Dysarthria	2 (4%)	1 (3%)	1 (7%)	2.23	0.15–32.98	0.53
Seizure	1 (2%)	0 (0%)	1 (7%)	–	–	–
Trace of tick bite	21 (44%)	19 (58%)	2 (13%)	0.38	0.11–1.30	0.05
Lymphadenopathy	19 (40%)	14 (42%)	5 (33%)	0.88	0.42–1.84	1.00
Bleeding tendency	10 (21%)	6 (18%)	4 (27%)			
Petechia	4 (8%)	3 (9%)	1 (7%)	0.72	0.08–6.27	1.00
Melena	2 (4%)	2 (6%)	0 (0%)	–	–	–
Gingival bleeding	1 (2%)	0 (0%)	1 (7%)	–	–	–
Arthralgia	9 (19%)	8 (24%)	1 (7%)	0.35	0.05–2.46	0.40
Headache	9 (19%)	8 (24%)	1 (7%)	0.31	0.04–2.17	0.24
Myalgia	8 (17%)	6 (18%)	2 (13%)	0.88	0.21–3.72	1.00
Hepatosplenomegaly	2 (4%)	1 (3%)	1 (7%)	2.08	0.14–30.65	1.00

Note: Abbreviations. RR, risk ratio; CI, confidence interval

*Fisher’s exact test

**Table 3:** Clinical characteristics of 48 case-patients with severe fever with thrombocytopenia syndrome during hospitalization grouped according to outcome in Japan.

	At admission and at any time during hospitalization					
	Total (n = 48)	Nonfatal (n = 33)	Fatal (n = 15)	RR	95% CI	p-value*
Temperature≥38°C	45 (94%)	31 (94%)	14 (93%)	0.99	0.85–1.17	1.00
Fatigue	40 (83%)	27 (82%)	13 (87%)	1.04	0.97–1.11	1.00
Gastrointestinal symptoms	42 (88%)	28 (85%)	14 (93%)			
Diarrhea	37 (77%)	25 (76%)	12 (80%)	1.13	0.85–1.51	0.70
Abdominal pain	19 (40%)	15 (45%)	4 (27%)	0.68	0.29–1.60	0.48
Nausea	17 (35%)	12 (36%)	5 (33%)	0.96	0.43–2.17	1.00
Neurological symptoms	34 (71%)	19 (58%)	15 (100%)			
Disorientation	31 (65%)	16 (48%)	15 (100%)	1.94	1.38–2.72	0.0007
Muscle weakness	10 (21%)	7 (21%)	3 (20%)	1.13	0.35–3.61	1.00
Tremor	10 (21%)	4 (12%)	6 (40%)	3.46	1.17–10.24	0.04
Dysarthria	6 (13%)	3 (9%)	3 (20%)	2.23	0.52–9.61	0.35
Seizure	8 (17%)	2 (6%)	6 (40%)	6.63	1.48–27.94	0.008
Lymphadenopathy	22 (46%)	17 (52%)	5 (33%)	0.69	0.33–1.43	0.32
Bleeding tendency	19 (40%)	11 (33%)	8 (53%)			
Petechia	16 (33%)	9 (27%)	8 (53%)	1.67	0.78–3.56	0.31
Melena	6 (13%)	4 (12%)	2 (13%)	1.04	0.21–4.99	1.00
Gingival bleeding	6 (13%)	3 (9%)	3 (20%)	2.31	0.53–9.96	0.35
Arthralgia	10 (21%)	9 (27%)	1 (7%)	0.36	0.05–2.46	0.40
Headache	9 (19%)	8 (24%)	1 (7%)	0.35	0.05–2.37	0.39
Myalgia	11 (23%)	9 (27%)	2 (13%)	0.64	0.17–2.49	0.70
Hepatosplenomegaly	6 (13%)	4 (12%)	2 (13%)	1.00	0.21–4.77	1.00

Note: Abbreviations. RR, risk ratio; CI, confidence interval

*Fisher’s exact test

Five of the 48 hospitalized case-patients (10%) had complications of fungal infections. Pulmonary aspergillosis and oral candidiasis were diagnosed in 3 and 2 case-patients, respectively. One case of proven invasive aspergillosis was documented in a histopathological study [15], and the other two cases were probable invasive aspergillosis. No obvious pulmonary symptom was found at the time of admission for these three case-patients. The case-patient with proven invasive aspergillosis had a fatal outcome; the others survived. Corticosteroids were used in 4 of the 5 case-patients with fungal infection and in 24 of the 43 case-patients without fungal infection. No corticosteroids were used in 20 case-patients: 1 patient with fungal infection and 19 patients without fungal infection. The use of corticosteroid did not differ significantly between hospitalized case-patients with or without fungal infection (p = 0.58).

### Laboratory Data

The laboratory findings of the 48 case-patients at admission are shown in Table 4. The median white blood cell (WBC) count and median platelet count were 1,725/μL (IQR: 1170–2668) and 6.3 × 10^4^/μL (IQR: 4.3–9.4), both of which were lower than the normal range. The platelet count differed between the fatal and nonfatal cases (p = 0.004), but the WBC count did not differ between groups. The median serum C-reactive protein concentration was 0.16 mg/dL (IQR: 0.05–0.50). The concentrations of serum aspartate aminotransferase (AST) and creatinine were significantly higher in the fatal than in the nonfatal cases.

**Table 4:** Laboratory findings of 48 case-patients with severe fever with thrombocytopenia syndrome at admission grouped according to outcome in Japan.

			Total (n = 48)			Non-fatal (n = 33)			Fatal (n = 15)			
		Normal range	n	Median	IQR	n	Median	IQR	n	Median	IQR	p-value
Hb	(g/dL)	M: 13.7–16.8F: 11.6–14.8	48	14.0	12.0–14.9	33	14.1	12.0–14.9	15	13.2	12.2–14.3	0.34
WBC	(/μL)	3300–8600	48	1725	1170–2668	33	1770	1400–2570	15	1290	965–2750	0.22
Platelet	(×10^4^/μL)	15.8–34.8	48	6.3	4.3–9.4	33	7.1	5.8–9.9	15	4.2	2.4–6.5	**0.004**
AST	(IU/L)	13–30	48	125	80–252	33	104	61–186	15	243	108–439	**0.02**
ALT	(IU/L)	M: 10–42F: 7–23	48	55	39–108	33	53	31–97	15	61	48–180	0.13
LDH	(IU/L)	124–222	47	431	351–908	32	406	328–659	15	831	402–1064	0.06
Albumin	(g/dL)	4.1–5.1	48	3.4	3.1–3.8	33	3.7	3.3–3.9	15	3.0	2.8–3.4	**0.02**
BUN	(mg/dL)	8–20	48	22	17–29	33	21	16–25	15	25.6	19.7–36.1	0.06
Cr	(mg/dL)	M: 0.65–1.07F: 0.46–0.79	48	0.93	0.70–1.13	33	0.82	0.70–1.07	15	1.12	0.98–1.38	**0.01**
CRP	(mg/dL)	0.00–0.14	47	0.16	0.05–0.50	32	0.14	0.05–0.36	15	0.27	0.04–0.78	0.35

Note: Abbreviations. n, number; IQR, interquartile range; M, male; F, female; Hb, hemoglobin; WBC, white blood cell; AST, aspartate aminotransferase; ALT, alanine aminotransferase; LDH, lactate dehydrogenase; BUN, blood urea nitrogen; Cr, creatinine; CRP, C-reactive protein

**Fig 1:**
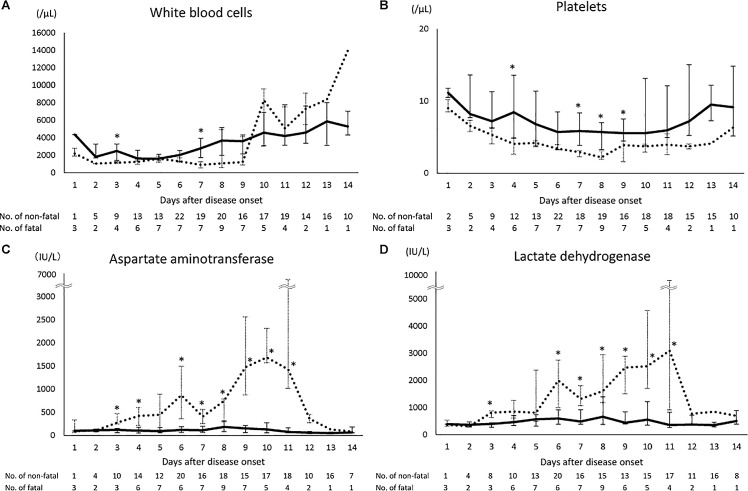
Laboratory findings for SFTS cases. White blood cells (A), platelets (B), aspartate aminotransferase concentration (C), and lactate dehydrogenase concentration (D) in nonfatal and fatal cases at the time of admission and during hospitalization. Laboratory data are shown as the median and interquartile range between the first and third quartiles. Solid lines indicate nonfatal cases and dotted lines indicate fatal cases. The numbers of nonfatal and fatal cases are indicated for each onset day below the graph. The normal ranges for each parameter are 3,500–9,000/μL for WBC count, 15–35 × 10^4/^μL for platelet count, 10–35 IU/L for AST, and 120–220 IU/L for serum LDH. * p < 0.05 between nonfatal and fatal cases.

**Fig 2:**
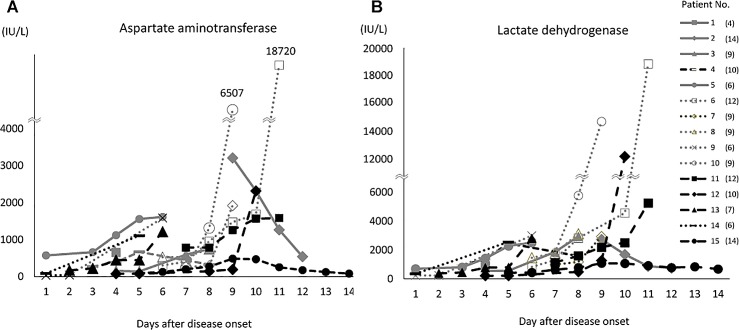
Serial concentrations of aspartate aminotransferase (A) and lactate dehydrogenase (B) in 15 fatal SFTS cases during hospitalization. The number in parenthesis of each patient indicates the days after disease onset when each patient died. A single result was available for the patient no.1. The normal ranges for each parameter are 10–35 IU/L for AST, and 120–220 IU/L for serum LDH.

The laboratory data of the case-patients at the time of admission and during hospitalization are shown in Fig 1. WBC count differed significantly between the fatal and nonfatal case-patients at days 3 and 7. WBC counts tended to be lower in the fatal case-patients than in the nonfatal case-patients until day 9 but to then increase rapidly after day 10 in the fatal case-patients. Platelet count was significantly lower in the fatal case-patients than in the nonfatal case-patients on days 4 and 7–9. The serum AST and lactate dehydrogenase (LDH) concentrations were elevated above the normal range during hospitalization in both the fatal and nonfatal case-patients. The serum concentrations were significantly higher in the fatal case-patients than in the nonfatal case-patients on days 3, 4, and 6–11 for AST and on days 3 and 6–11 for LDH. In addition, serial concentrations of serum AST and LDH for fifteen fatal case-patients are shown in Fig 2. The serum AST and LDH concentrations progressively increased in nine (60%) and ten (67%) of these case-patients during their clinical course, respectively. Especially in two case-patients, the concentrations of these enzymes dramatically increased before their deaths. Hemophagocytosis was observed in 15 of the 18 case-patients (83%) whose bone marrow samples were tested for pathology.

## Discussion

The incidence of SFTS (0.05 cases/100,000 person-years) found in Japan is similar to that in South Korea (0.07 cases/100,000 person-years) [16] but much lower than that in China (0.12–0.73/100,000 person-years) [17]. In our retrospective study, the median age of the 49 included patients was 78 years, which was much higher than in the reports from China (50–60 years) [18,19] and South Korea (69 years) [16]. The current median age of the whole population is higher in Japan (45.9 years) than in China (35.4 years) and South Korea (39.4 years) [20]. In addition, one fourth of the Japanese population was older than 65 years of age in 2013 [21]. Therefore, the findings of the present study may reflect the higher proportion of aged people among the population in Japan.

SFTS cases have been detected between the spring and summer in Japan. A similar seasonality was found in China [3,19] and South Korea [16]. A study from South Korea reported that the infection rate of SFTSV among ticks was higher in nymphs and adults [7]. Another study demonstrated that SFTSV was detected in ticks collected between April and September in South Korea [22]. Because human rural activities, such as hiking and camping, tend to increase from spring to summer [3], these two factors, the nature of ticks and human activities, may explain the seasonality of SFTS infection in humans.

Most case-patients were retired or unemployed, or farmers, and undertook outdoor activities such as farming or plant collection within 2 weeks before the onset of illness. Collectively, our data suggest these aged people in the western region of Japan experienced tick bites during their latest outdoor activities and consequently acquired SFTSV infection. This distribution is consistent with that for *A*. *testudinarium* in Japan [23], which suggests that *A*. *testudinarium* is one possible vector of SFTSV in Japan. Because *H*. *longicornis* was found widely in Japan [24], SFTS cases may emerge in the eastern regions of Japan in the future.

The case-fatality proportion (31%) in our study is higher than the 7.3–12.2% in recent reports from China [3,25], and lower than the 46% reported from South Korea [16]. Several reasons may explain the high case-fatality proportion in Japan. First, most of the patients in Japan were of advanced age, and two thirds of them had underlying diseases. Second, the interim case definition might have biased the reported cases toward clinically severe conditions [12]. Although serological assays were available for laboratory diagnosis in some local health laboratories, no case was diagnosed by serological assays in this study. Third, the lack of serological testing may have failed to detect mild cases in our analysis, although a mild case of SFTS has been reported in Japan [26].

It was demonstrated that most of the SFTSV isolated from Japanese and Chinese cases formed independent clades, Japanese clade consisted of genotypes J1, J2, J3 and Chinese clade consisted of genotypes C1 to C5, respectively [27]. However, some SFTSV isolated from Japanese cases belonged to Chinese clade (genotypes C4 and C5), while the SFTSV isolated from Chinese cases belonged to the Japanese clade (J3). Amino acid homology in RNA-dependent RNA polymerase, glycoprotein, and nucleocapsid protein within the Japanese genotypes were over 98%, suggesting that there seems to be no association between severity of the disease and the genotypes.

About 50% of the patients exhibited neurological abnormalities at admission, and significant difference was found in neurological findings during hospitalization between the fatal and nonfatal cases. A previous study also reported that the presence of neurological manifestations was an independent predictor of the risk for severe disease in Northeast China [25]. We also found significant increases of the serum AST and LDH concentrations in the fatal case-patients during hospitalization, but the increases of these serum enzymes were not found in all fatal case-patients in this study. However, the serum AST and LDH concentrations may be important biomarkers of clinical outcome, which is in agreement with previous reports [28,29].

Interestingly, fungal infections such as invasive aspergillosis were found in 10% of these case-patients. We found no significant effects of treatment with corticosteroids on the complications of fungal infections among our case-patients. Decreases in WBC and CD4 T-cell counts have been reported during the acute phase of SFTS [30], and it is possible that the complication of invasive fungal infection observed in our case-patients may be explained, at least in part, by SFTSV-induced immunosuppression during the acute phase.

Although it has been suggested that SFTSV-induced thrombocytopenia is caused by the clearance of circulating virus-bound platelets by splenic macrophages in a mouse model [31], no statistical difference was found in the platelet counts between 15 cases with hemophagocytosis and three cases without hemophagocytosis.

None of the treatments, including ribavirin for SFTS, appears to be effective [29]. However, favipiravir, which was used in humans as an antiviral agent against influenza virus [32], has been reported to be effective against SFTSV infection in a murine model [33]. The effect of favipiravir in the treatment of SFTSV infections using an interferon alpha receptor knockout mouse model was demonstrated when it was administered within 5 days postinfection, while the median time from onset to death of fatal cases was 8 days in our cases. Favipiravir is expected to have an efficacy in the treatment of SFTS; however, to evaluate this, a clinical study in which favipiravir should be administered to the case-patients exhibiting clinical predictors such as neurological abnormalities as soon as possible, is required.

This study has several limitations. First, 49 (51%) of the 96 reported cases were included in this study, but they may not represent the population of interest. Second, the case-fatality proportion might have been overestimated, as discussed above. Third, we cannot exclude the effects of confounding factors on the risk of death because of the limited number of case-patients. Further studies, including case–control studies, are required to identify the risk factors for SFTSV infection in Japan.

In conclusion, most confirmed cases of SFTS reported between April and August in 2013 and 2014 were found in the western region of Japan. Most of the case-patients were of advanced age, and the case-fatality proportion was 31%. Most case-patients had a history of outdoor activity within 2 weeks before the onset of illness. Local residents should try to avoid tick bites during their outdoor activities. The high percentage of patients with neurological abnormalities in the fatal cases during hospitalization suggests that these clinical findings may be useful for clinicians to monitor and predict the prognosis of SFTS.

## Ethical Approval

The research protocol was approved by the research and ethics committees of NIID (no. 549). This study was carried out according to the principles expressed in the Declaration of Helsinki. The patient or guardian provided written informed consent for all cases.

## Supporting Information

S1 Fig Geographic distribution of 96 cases of SFTS reported to the National Epidemiological Surveillance of Infectious Diseases in Japan between March 2013 and September 2014. The prefectures where the cases were reported are shown in gray.
(PPTX)

S2 Fig Epidemic curve of 96 cases of SFTS reported to the National Epidemiological Surveillance of Infectious Diseases in Japan between March 2013 and September 2014. (PPTX)

S1 Table Geographical distribution of 96 cases of severe fever with thrombocytopenia syndrome reported to National Epidemiological Surveillance of Infectious Diseases. (DOCX)

S2 Table Basic characteristics of 96 case-patients with severe fever with thrombocytopenia syndrome reported to National Epidemiological Surveillance of Infectious Diseases. (DOCX)
