# Supplementary material for: Epidemiological and Clinical Features of Severe Fever with Thrombocytopenia Syndrome in Japan, 2013–2014
Source: PLoS One. 2016 Oct 24;11(10):e0165207. doi: 10.1371/journal.pone.0165207 (PMC5077122; doi:10.1371/journal.pone.0165207)
Supplement: S1 Fig — The prefectures where the cases were reported are shown in gray. (PPTX) [file pone.0165207.s001.pptx]

## Slide 1
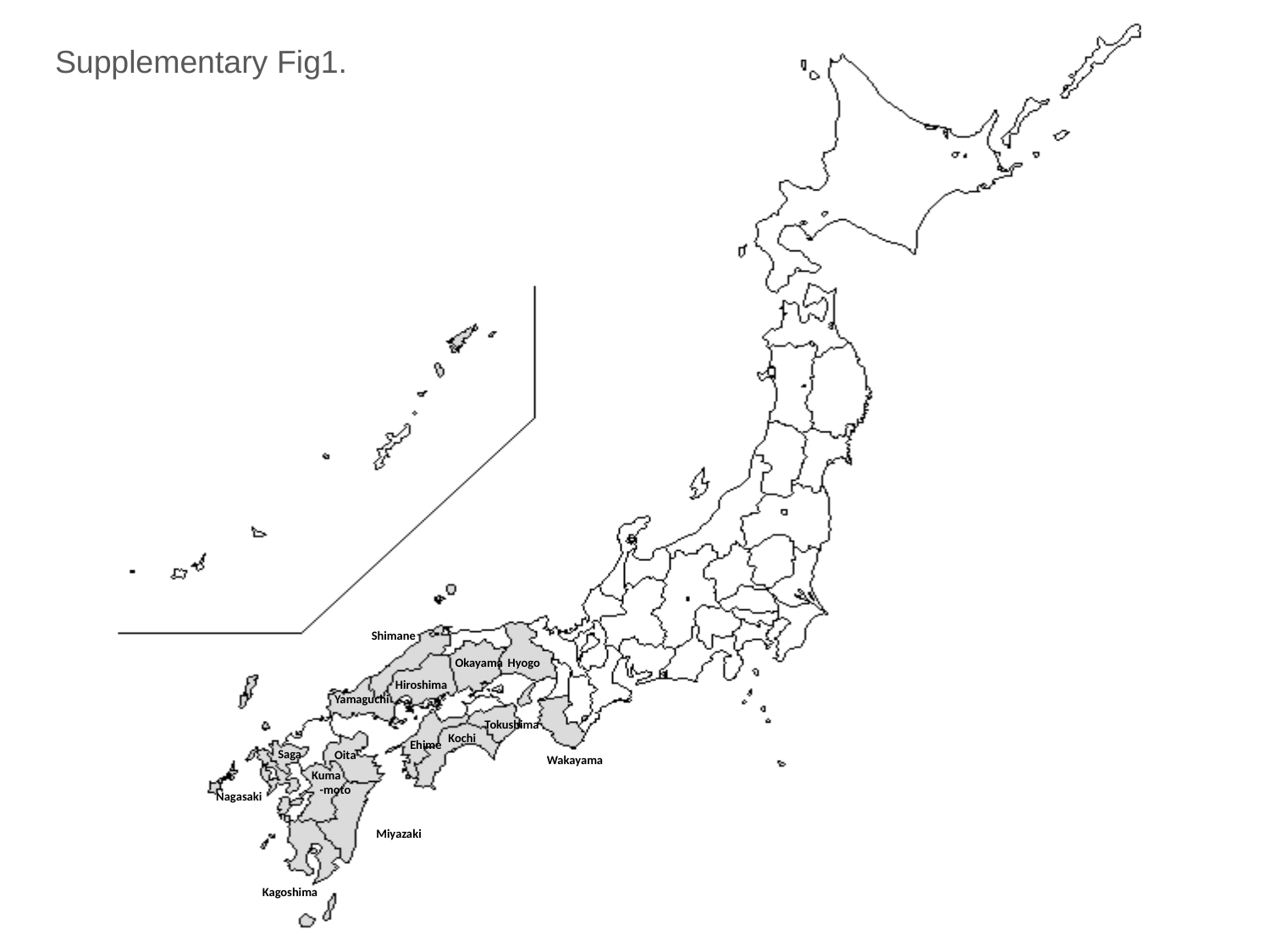

Supplementary Fig1.
Shimane
Okayama
Hyogo
Hiroshima
Yamaguchi
Tokushima
Kochi
Ehime
Saga
Oita
Wakayama
Kuma
 -moto
Nagasaki
Miyazaki
Kagoshima
