# Supplementary material for: Epidemiological and Clinical Features of Severe Fever with Thrombocytopenia Syndrome in Japan, 2013–2014
Source: PLoS One. 2016 Oct 24;11(10):e0165207. doi: 10.1371/journal.pone.0165207 (PMC5077122; doi:10.1371/journal.pone.0165207)
Supplement: S2 Fig — (PPTX) [file pone.0165207.s002.pptx]

## Slide 1
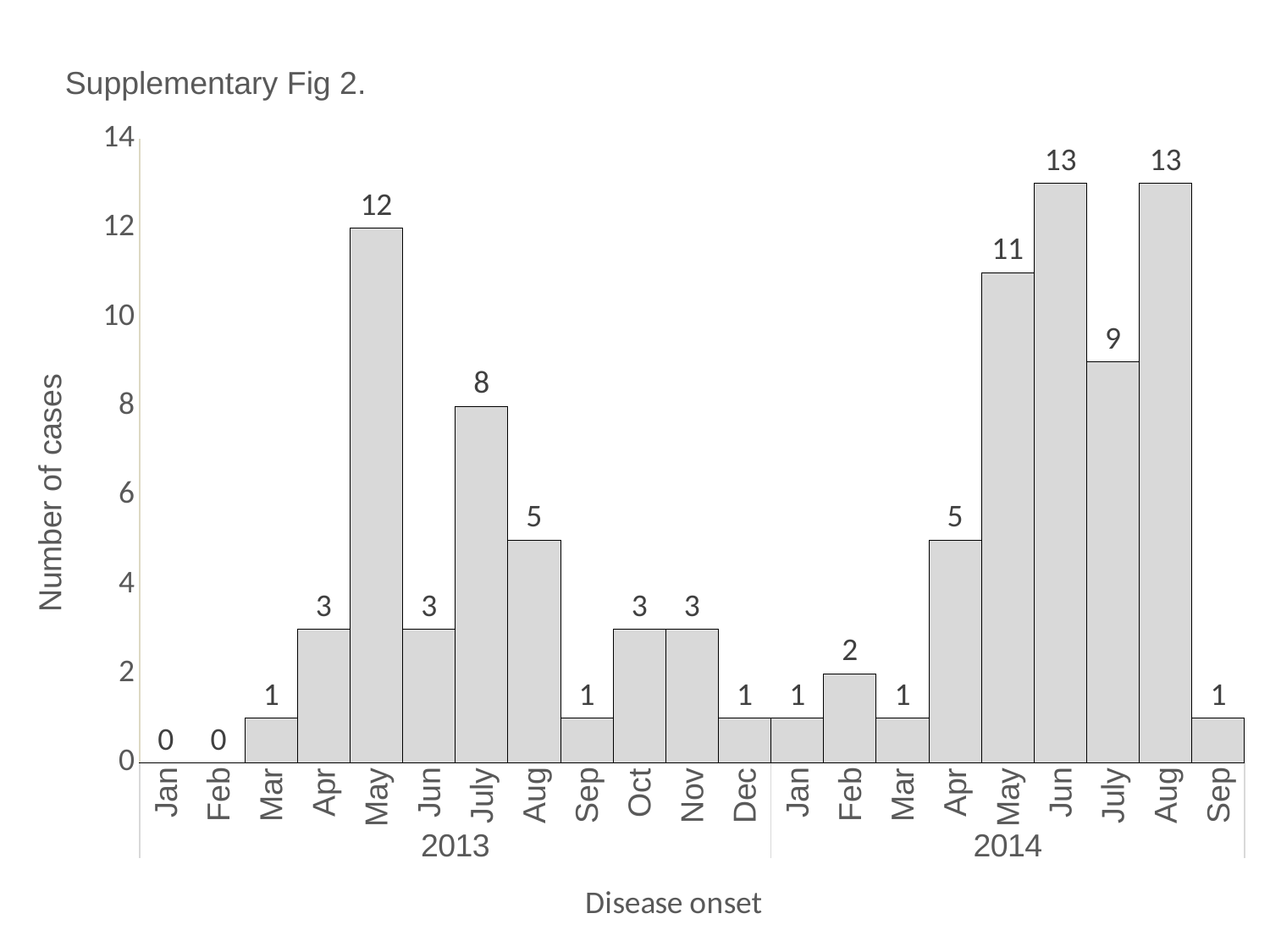

Supplementary Fig 2.
### Chart
| Category | |
|---|---|
| Jan | 0.0 |
| Feb | 0.0 |
| Mar | 1.0 |
| Apr | 3.0 |
| May | 12.0 |
| Jun | 3.0 |
| July | 8.0 |
| Aug | 5.0 |
| Sep | 1.0 |
| Oct | 3.0 |
| Nov | 3.0 |
| Dec | 1.0 |
| Jan | 1.0 |
| Feb | 2.0 |
| Mar | 1.0 |
| Apr | 5.0 |
| May | 11.0 |
| Jun | 13.0 |
| July | 9.0 |
| Aug | 13.0 |
| Sep | 1.0 |
