# Supplementary material for: Epidemiological and Clinical Features of Severe Fever with Thrombocytopenia Syndrome in Japan, 2013–2014
Source: PLoS One. 2016 Oct 24;11(10):e0165207. doi: 10.1371/journal.pone.0165207 (PMC5077122; doi:10.1371/journal.pone.0165207)
Supplement: S1 Table — (DOCX) [file pone.0165207.s003.docx]

**S1 Table.** **Geographical distribution of 96 cases of severe fever with thrombocytopenia syndrome reported to the National Epidemiological Surveillance of Infectious Diseases**

|  | Total number (n = 96) |
| --- | --- |
| Kinki region |  |
| Hyogo | 2 (2.1%) |
| Wakayama | 2 (2.1%) |
| Chugoku region |  |
| Okayama | 4 (4.2%) |
| Hiroshima | 7 (7.3%) |
| Yamaguchi | 6 (6.3%) |
| Shimane | 1 (1.0%) |
| Shikoku region |  |
| Tokushima | 9 (9.4%) |
| Ehime | 17 (17.7%) |
| Kochi | 10 (10.4%) |
| Kyusyu region |  |
| Saga | 1 (1.0%) |
| Nagasaki | 5 (5.2%) |
| Kumamoto | 4 (4.2%) |
| Oita | 1 (1.0%) |
| Miyazaki | 18 (18.8%) |
| Kagoshima | 9 (9.4%) |
