# Supplementary material for: Epidemiological and Clinical Features of Severe Fever with Thrombocytopenia Syndrome in Japan, 2013–2014
Source: PLoS One. 2016 Oct 24;11(10):e0165207. doi: 10.1371/journal.pone.0165207 (PMC5077122; doi:10.1371/journal.pone.0165207)
Supplement: S2 Table — (DOCX) [file pone.0165207.s004.docx]

**S2 Table.** **Basic characteristics of 96 case-patients with severe fever with thrombocytopenia syndrome reported to the National Epidemiological Surveillance of Infectious Diseases**

|  |  | Total number (n = 96) |
| --- | --- | --- |
| Sex | |  |
| Male | | 41 (43%) |
| Female | | 55 (57%) |
| Age, y [IQR] | | 74 [63–83] |
| <40 | | 2 (2%) |
| 40–49 | | 2 (2%) |
| 50–59 | | 7 (7%) |
| 60–69 | | 24 (25%) |
| 70–79 | | 28 (29%) |
| 80–89 | | 28 (29%) |
| 90–99 | | 5 (5%) |
